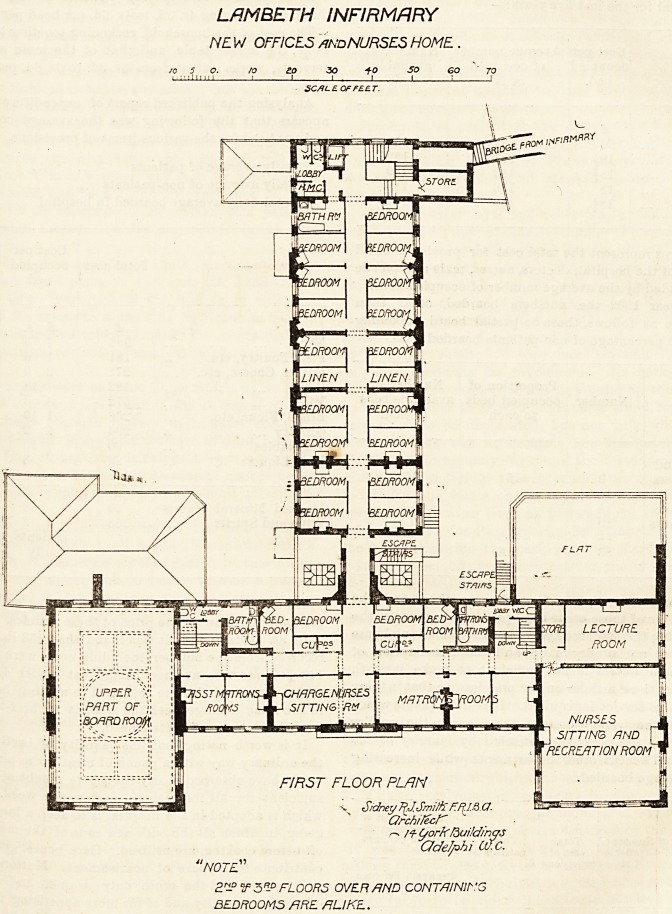# New Offices and Nurses' Home at the Lambeth Infirmary, Brook Street

**Published:** 1904-05-14

**Authors:** 


### HOSPITAL ADMINISTRATION.

### CONSTRUCTION AND ECONOMICS.

### NEW OFFICES AND NURSES' HOME AT THE LAMBETH INFIRMARY, BROOK STREET.

THIS work comprises the reconstruction of the offices and a new home for the nurses. The whole building is very conveniently arranged, and the details have been most carefully planned and carried out; and it shows that these parochial infirmaries are moving steadily onwards in the matter of suitable provision for their nurses, on which so much of the welfare of the whole institution depends.

The block is somewhat in shape like the letter T, the horizontal part of the letter facing north; and its position is between and in close proximity to the ward blocks. The first floor of the nurses' home communicates by a bridge with the wards.

The main elevation is to the north, and in its centre are the ntrance, the vestibule and an octagonal hall. On the right on entering are the clerks' office, the private office, and the chief part of the right wing to the north is taken up by the general office, behind which are various sub-offices and a large lavatory for the clerks' use. On the left of the entrance are the waiting-room, the relief committee-room, and the board-room with its committee-room, waiting-room and lavatories. This left wing, of course, balances the right wing—and the elevation ought to look well in the style of architecture adopted. Behind the central offices runs a corridor, or rather two short corridors run and meet in the octagonal hall. On one side is the lady guardians' room, and on the other is the hall porter's room; between which is a lobby giving nurses' sitting-room and lecture-room. Here are also the matron's rooms, and those of the assistant-matron, and various bedrooms and offices.

The space over the kitchen department is divided longitudinally by a corridor, and on either side of this are the nurses' bedrooms, there being 17 of these. The corridor is quite 70 feet long, and is lighted by windows at its south access to the superintendent relieving officer's room, and the lunacy warrant officer's room. On the south of these and occupying the vertical limb of the letter are the kitchen with its offices, the nurses' dining-room, cloak-room, lift, staircase and entrance hall. A visitors' room opens from this south entrance, and adjoining it is a room for bicycles.

On the first floor and over the general office are the end. There are three floors given up to the nursing staff, each nurse having a room, and there is accommodation for 100 nurses.

The buildings are carried out in Georgian style, and the material is red brick with stone facings.

The architect is Mr. Sidney Smith; the contractors are Messrs. Lawrence and Son, of Waltham Cross, and the clerk of the works is Mr. Nightingale.

## Figures and Tables

**Figure f1:**
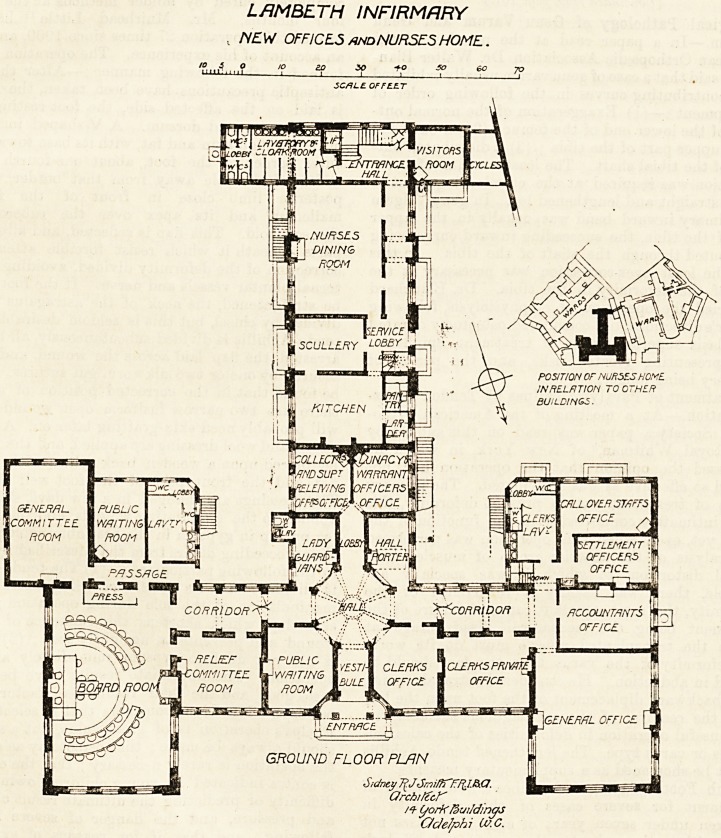


**Figure f2:**